# Prognostic significance of low DICER expression regulated by miR-130a in cervical cancer

**DOI:** 10.1038/cddis.2014.127

**Published:** 2014-05-01

**Authors:** L He, H-Y Wang, L Zhang, L Huang, J-D Li, Y Xiong, M-Y Zhang, W-H Jia, J-P Yun, R-Z Luo, M Zheng

**Affiliations:** 1 State Key Laboratory of Oncology in Southern China, Sun Yat-Sen University Cancer Center, 651 Dongfeng Road East, Guangzhou 510060, China; 2 Department of Gynecology, Sun Yat-Sen University Cancer Center, 651 Dongfeng Road East, Guangzhou 510060, China; 3 Department of Obstetrics and Gynecology, The First Affiliated Hospital, Sun Yat-sen University, Guangzhou, China; 4 Department of Pathology, Sun Yat-Sen University Cancer Center, 651 Dongfeng Road East, Guangzhou 510060, China

**Keywords:** Dicer, miR-130, cervical cancer, microRNA, prognostic factor

## Abstract

Dicer is crucial for the maturation of microRNAs (miRNAs) and its dysregulation may contribute to tumor initiation and progression. The study explored the clinical implications of Dicer and its post-transcriptional regulation by microRNAs in cervical cancer. qRT-PCR and immunohistochemistry investigated Dicer mRNA and protein levels in cervical cancer tissues. The relationship between Dicer expression and survival was analyzed. MiRNA target prediction identified miRNAs that might target Dicer. Luciferase reporter and gain- or loss-of-function assays were performed. The results showed that 36.7% of cervical cancer cases showed low expression of Dicer mRNA and 63.3% cases showed high expression. At the protein level, 51% cases showed negative expression and 49% cases showed positive expression. Dicer mRNA and protein expressions were significantly associated with distant metastasis and recurrence in cervical cancer (*P*=0.002 and *P*=0.012, respectively). Multivariate Cox analysis indicated that low Dicer expression (*P*=0.016) and tumor stage (*P*=0.047) were independent predictors. Among the miRNAs predicted to target Dicer, 10 were detected by RT-PCR; their expressions were significantly higher in cervical cancers with lower Dicer expression than in those with higher Dicer expression and were negatively correlated with Dicer expression level (*P*<0.05). *In vitro* experiments demonstrated that miR-130a directly targeted Dicer mRNA to enhance migration and invasion in SiHa cells. Finally, survival analysis indicated that higher expression of miR-130a was significantly associated with poor disease-free survival. Taken together, Dicer expression regulated by miR-130a is an important potential prognostic factor in cervical cancer.

Dicer is a cytoplasmic RNase III enzyme that cleaves the loop of the pri-miRNA and long double-stranded RNA into ∼22 bp double-stranded miRNA and short interfering RNA (siRNA) that target specific messenger RNAs, resulting in gene silencing. Dicer has an important role in the regulation of cell number and in controlling apoptosis.^1, 2, 3^ Loss of Dicer in mice disrupts embryonic stem-cell differentiation and is lethal during early development.^4^ Low Dicer expression is associated with worse clinical outcomes in lung cancer,^5^ breast cancer,^6^ and endometrial adenocarcinoma.^7^ Low expression of Dicer and Drosha is associated with ovarian cancer progression and poor clinical outcomes.^8^

MiRNAs are small non-coding RNAs that modulate gene expression, mainly by base pairing to the 3′-untranslated region (UTR) of the target mRNA post-transcriptionally.^9^ They are predicted to control >60% of human genes.^10^ They have important roles in development, cellular differentiation, proliferation, cell cycle control and cell death,^11^ and have been implicated in a variety of human diseases, including cancer.^11, 12^ For example, miRNA-130a antagonizes the inhibitory effects of GAX on endothelial cell proliferation, migration, and the inhibitory effects of HoxA5 on tube formation *in vitro.*^13^ MiRNA-130a overexpression was associated with lymph node metastasis and poor prognosis of non-small cell lung cancer (NSCLC).^14^ Higher expression of MiRNA-103/107, which attenuates miRNA biosynthesis by targeting and inhibiting Dicer, causes global miRNA downregulation and acts as a prognostic marker in breast tumors.^15^ Dicer-targeting miRNAs regulate Dicer expression and constitute a negative feedback loop.^16^ There is increasing evidence that the expression of miRNA genes is aberrant in cervical cancer, and a subset of miRNAs is identified that correlate with disease stage and recurrence.^17, 18, 19^ Till now, there has been no study on Dicer expression in cervical carcinoma.

In this study, we investigated Dicer expression in a large cohort of primary invasive cervical carcinomas. Using an *in silico* approach, we identified several conserved miRNAs predicted to target the 3′-UTR of Dicer. Then, we investigated the role of miRNAs in the regulation of Dicer expression and revealed the upstream regulation mechanism. Finally, we explored the function of miR-130a in cervical cancer cell lines.

## Results

### Expression of Dicer in cervical carcinoma

**Figure 1:**
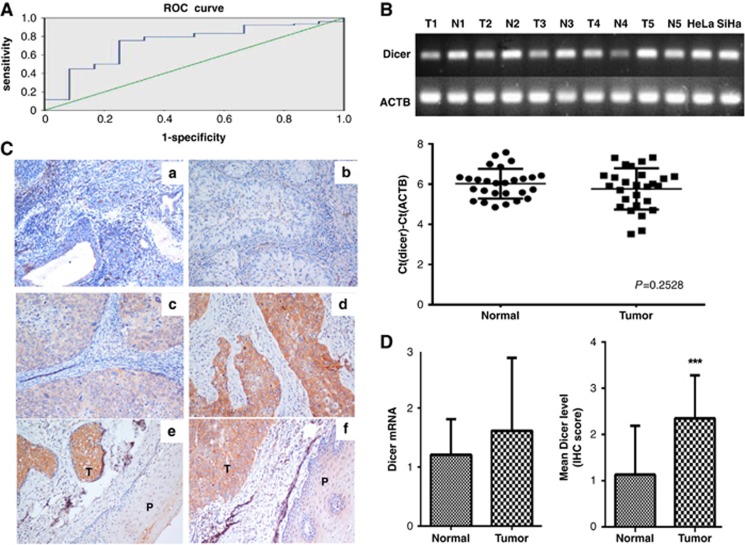
(**A**) A ROC curve was used to discriminate two distinct groups of Dicer expression. (**B**) The mRNA expression of Dicer was not significantly different between cervical cancer tissues and matched adjacent non-cancerous tissues. (**C**) Dicer protein levels in cervical cancer tissues and adjacent non-cancerous tissues. (a) Cervical squamous cell cancer, rated Dicer (0) ( × 200). (b) Cervical adenocarcinoma, rated Dicer (1) ( × 200). (c) Cervical squamous cell cancer, rated Dicer (2) ( × 200). (d) Cervical squamous cell cancer, rated Dicer (3) ( × 200). (e, f) Immunohistochemistry showing high expression of Dicer in cervical squamous cell cancer, and low expression of Dicer in adjacent non-cancerous tissues. t, tumor; p, adjacent non-cancerous tissues. (**D**) Dicer protein expression in the cervical cancer samples was much higher than that in the matched adjacent non-cancerous samples

We detected Dicer mRNA levels in 90 cervical cancer tissues and 23 adjacent non-cancerous tissues using qRT-PCR. The Dicer mRNA levels in the cervical cancer tissues were not normally distributed (*P*=0.013 by the Kolmogorov–Smirnov test for normality). A histogram of Dicer expression showed a frequency distribution with two prominent peaks at log2 values from −1.0 to −0.67 and from 0 to 0.33. A ROC curve was therefore used to identify the cut-off value with the highest potential for discriminating two distinct groups in terms of the log2 ratio of Dicer expression (*P*=0.007) (Figure 1A). The value was found to be −0.0192, which was close to its mean values (0.15). Dicer mRNA expression varied among cervical cancer specimens. Low Dicer expression was observed in 36.7% of samples; high Dicer mRNA expression was observed in 63.3% of samples. The median ratio of Dicer expression in cancer specimens with low Dicer mRNA expression was 0.578 (range, 0.18–0.99); specimens with high Dicer mRNA expression had a median ratio of 1.47 (range, 0.99–6.03). To determine whether Dicer mRNA levels reflected protein expression, 102 cervical cancers specimens, including samples from the same 90 cases detected by qRT-PCR, were also examined using immunohistochemistry (IHC) (Table 1). Fifty-two cases (51%) were negative for Dicer expression, whereas 50 cases (49%) were positive for Dicer. The IHC score agreed with the qRT-PCR results for Dicer (kappa=0.717; 95% confidence interval (CI), 0.704–0.853). In the 23 pairs of samples, the relative mRNA expression of Dicer was not significantly different between cervical cancer tissues and the matched adjacent non-cancerous tissues (*P*=0.2528) (Figures 1B and D). However, IHC analysis showed that Dicer protein expression in the cervical cancer samples was much higher than in the matched adjacent non-cancerous samples (*n*=23, *P*<0.05) (Figures 1C and D). The inconsistency between Dicer mRNA and protein level in cervical cancer indicated that a post-transcriptional mechanism is involved in regulating Dicer expression in cervical cancer.

### Low expression of Dicer was associated with clinical stage and recurrence

**Figure 2:**
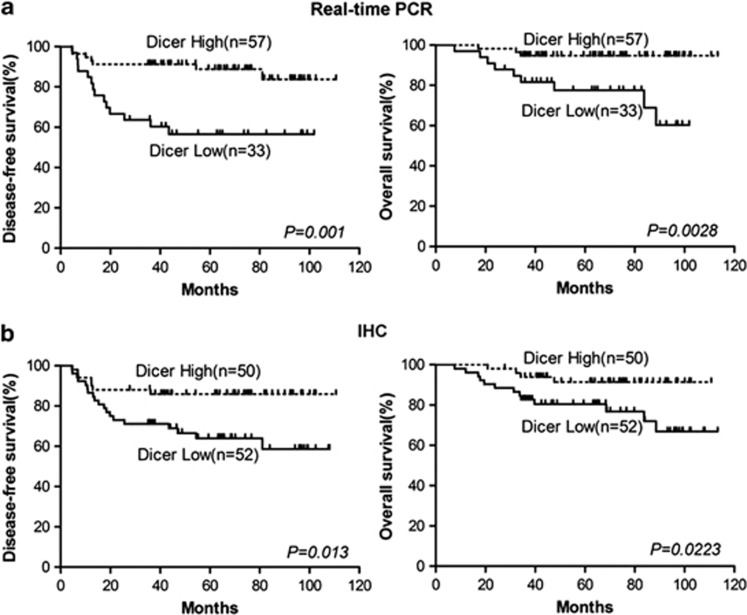
Cervical cancer patients with low Dicer expression had a significantly shorter disease-free survival and overall survival than those with high expression of Dicer at both the mRNA (**a**) and protein levels (**b**)

We then analyzed the relationships between Dicer expression level (mRNA and protein) and the clinical characteristics of 102 patients with invasive cervical carcinoma. Chi-square tests showed that neither Dicer mRNA nor Dicer protein level was significantly associated with age, tumor grade, histology, tumor size, lymph node metastasis or squamous cell carcinoma antigen (SCC) level (Table 1). However, Dicer protein expression was significantly associated with advanced tumor stage (*P*=0.015). Both mRNA and protein expression levels were significantly associated with recurrence, including distant metastasis and local recurrence (*P*=0.002 and *P*=0.012, respectively). Kaplan–Meier survival analyses showed that cervical cancer patients with low Dicer mRNA expression had a significantly shorter 5-year disease-free survival (DFS) (57.6% *versus* 87.7%, *P*=0.001) and 5-year overall survival (OS) (72.7% *versus* 94.7%, *P*=0.0028) than those with high Dicer mRNA expression (Figure 2a). Similarly, patients with low Dicer protein expression were significantly associated with shorter 5-year DFS (63.5% *versus* 86.0%, *P*=0.013) and 5-year OS (75% *versus* 92%, *P*=0.0223) than patients with high Dicer protein expression (Figure 2b). Furthermore, univariate Cox regression analysis of various parameters with OS showed that low expression of Dicer (protein hazard ratio (HR): 3.418, *P*=0.032; mRNA HR: 5.794, *P*=0.008) and clinical stage (HR: 7.761, *P*=0.01) were significant predictive factors for poor outcome. Similar results were observed between DFS and Dicer expression. Multivariate Cox regression showed that low expression of Dicer (*P*=0.016) and tumor stage (*P*=0.047) remained independent predictors (Table 2).

### Low Dicer expression in cervical cancers is inversely correlated with high expression of 10 miRNAs

**Figure 3:**
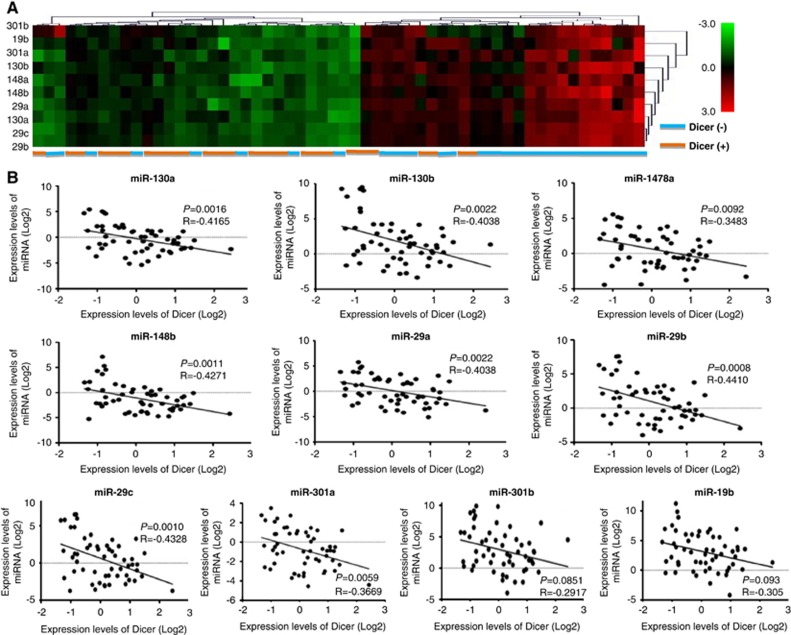
(**a**) Clustering analysis showing that the mRNA level of microRNAs in cervical cancer was negatively correlated with the Dicer mRNA expression level. (**b**) Correlation between Dicer and miRNA expressions in cervical cancer

We reasoned that the reduced expression of Dicer in cervical cancer might be induced by high expression of miRNAs. Using several miRNA target prediction programs, we found 10 miRNAs (hsa-miR-130a, -130b, -148a, -148b, -29a, -29b, -29c, -19b, -301a, and -301b) that were predicted to target the 3′-UTR of Dicer transcripts. The expression levels of these 10 miRNAs in 26 cervical cancer tissues with low Dicer expression and 29 with high Dicer expression were examined by qRT-PCR. The expression levels of the 10 miRNAs were all significantly higher in the low-Dicer-expression cervical cancers compared with the high-Dicer-expression cervical cancers (*P*<0.05) (Figure 3a). There was a significant inverse correlation between the expressions of Dicer and the 10 miRNAs in cervical cancer, with Pearson correlation coefficients ranging from −0.29 to −0.44 (Figure 3b). The data suggested that these miRNAs might target Dicer mRNA.

The relative mRNA expression of Dicer and the 10 miRNAs was determined by qRT-PCR on 55 cervical cancer tissues, 23 adjacent non-cancerous tissues and cervical cancer cells. Data revealed that the Dicer mRNA expression in cancer cells was higher than in the cancer tissues and adjacent non-cancerous tissues (3.05-fold and 2.18-fold, respectively). The expression levels of five miRNAs (miR-130a, -148b, -29a, -29c, and -301a) were significantly downregulated in cervical cancer tissues compared with adjacent non-cancerous tissues. Notably, among 10 miRNAs, miRNA-130a and miRNA-148b exhibited lower expression levels (by 3-fold) in cervical cancers compared with normal cervical tissues. Synthetic mimics of miRNA-130a and miRNA-148b were transfected individually into HeLa cells. qRT-PCR and western blotting results showed that overexpression of miRNA-130a could effectively downregulate Dicer expression to a greater extent than miRNA-148b (see Supplementary Figure 1).

### MiR-130a directly targets Dicer in cervical cancer cells

Out of the 10 miRNAs, miR-130a had the strongest relationship with Dicer and was predicted to target Dicer by most of the programs. This prompted us to focus on whether and how miR-130a targeted Dicer. To test the repressive potential of miRNA-130a on Dicer expression, a synthetic mimic of miRNA-130a was transfected into SiHa cells, and qRT-PCR and western blotting were used to monitor endogenous Dicer expression. Cells were also transfected with a scrambled mimic. The results showed that overexpression of miRNA-130a could effectively downregulate Dicer expression compared with the scrambled mimic. This indicated that miR-130a could inhibit Dicer expression.

**Figure 4:**
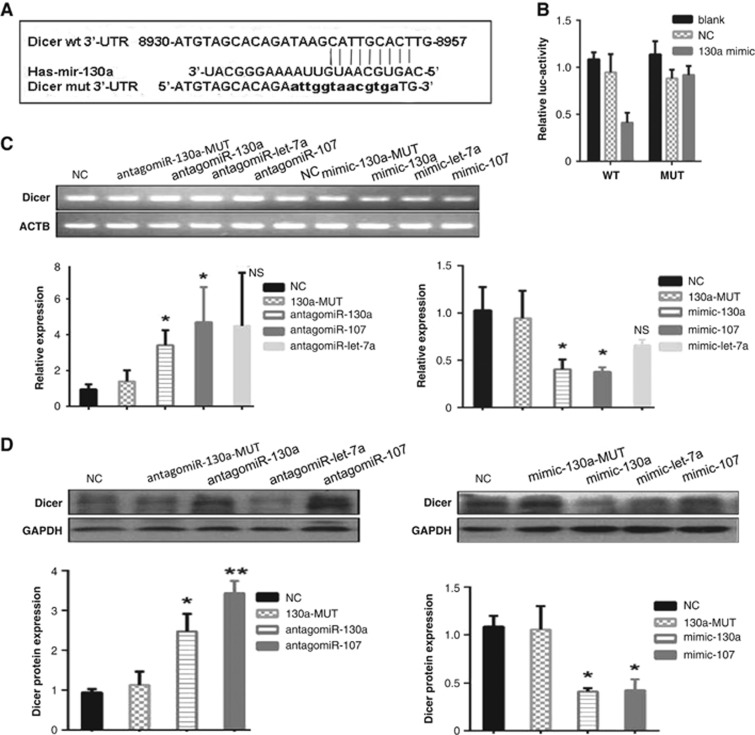
(**a**) Schematic illustration showing locations of the putative miR-130a target site and luciferase insert (Luc. insert) in the 3′-UTR of Dicer. The sequence alignment of mature miR-130a, its target site and mutated nucleotides are also shown. (**b**) Relative luciferase activity was measured using 293T cells and showed marked reduction following insertion of the 3′-UTR of wild-type (wt) Dicer, but not with the insertion of the 3′-UTR of the mutant (mut) Dicer. That reduction was significantly recovered following treatment with mimic-130a, but not with the negative control. (**c**) qRT-PCR analysis of expression level of dicer in SiHa cells transfected with antagomiRs and mimics. The mRNA level of Dicer in cervical cancer cell was upregulated by transfecting with antagomiR-130a and downregulated by transfecting with mimic-130a. (**d**) The protein level of Dicer was upregulated by antagomiRs (130a and 107) and downregulated by mimics (130a and 107); NC, negative control. We analyzed all *versus* control, except miR-130a, which we analyzed *versus* miR-130a-mut. *P<0.05 when compared with NC; **P<0.01 when compared with NC; NS means no significance when compared with NC.

To test whether miR-130a targeted Dicer directly, a luciferase reporter assay was performed. A dual-luciferase reporter vector containing the wild-type (Figure 4a) Dicer 3′-UTR was co-transfected into 293T cell with miR-130a mimics, scrabbled sequences (as a negative control, NC) or transfection reagents (as a blank control, BC). As expected, relative luciferase activity in 293T cells transfected with miR-130a mimics was significantly lower than that in the cells with scrabbled sequences or reagents (Figure 4b). To confirm that the reduced luciferase activity was specifically caused by miR-130a binding to the seed site of 3′-UTR, the seed sequence Dicer 3′-UTR was mutated in the luciferase reporter construct (Figure 4a). The result showed that luciferase activity in the cells containing the mutant reporter construct was the same when treated with miR-130a mimics, scrabbled sequences or transfection reagents. These results demonstrated that miR-130a could bind directly to Dicer 3′-UTR (Figure 4b).

We then asked whether miR-130a could suppress Dicer mRNA expression by binding to 3′-UTR of Dicer. Previous studies showed that let-7a^16, 20^ and miR-107^21^ also targeted Dicer. Therefore, to compare the inhibitory effect of miR-130a on Dicer expression with that of let-7a and miR-107, we transiently transfected SiHa cells with the three miRNA mimics, separately. To further verify that miR-130a targeted the Dicer 3′-UTR specifically, we also transfected miR-130a mutants into SiHa cells. qRT-PCR analysis showed that compared with NC, Dicer mRNA levels were reduced significantly by miR-130a and miR-107 (both *P*<0.05) and marginally significantly by let-7a (*P*=0.0622), but not by miR-130a-MUT (Figure 4c). To further confirm that the downregulated Dicer mRNA was caused specifically by these miRNAs, antagomiRs against these three miRNAs were transfected into SiHa cells. As shown in Figure 4c, the Dicer mRNA expression level was significantly increased in the cells with antagomiR-130a or antagomiR-107 (both *P*<0.05) and increased marginally significantly in cells with antagomiR let-7a, compared with cells with NC. Simultaneously, we also measured Dicer protein expression in SiHa cells transfected with these miRNA mimics and antagomiRs using western blotting. As shown in Figure 4d, similar results to the qRT-PCR experiment were observed. Taken together, these results indicated that miR-130a could target Dicer mRNA and markedly inhibited Dicer expression, comparably with miR-107 and stronger than let-7a.

### MiR-130a promotes migration and invasion of SiHa cells

**Figure 5:**
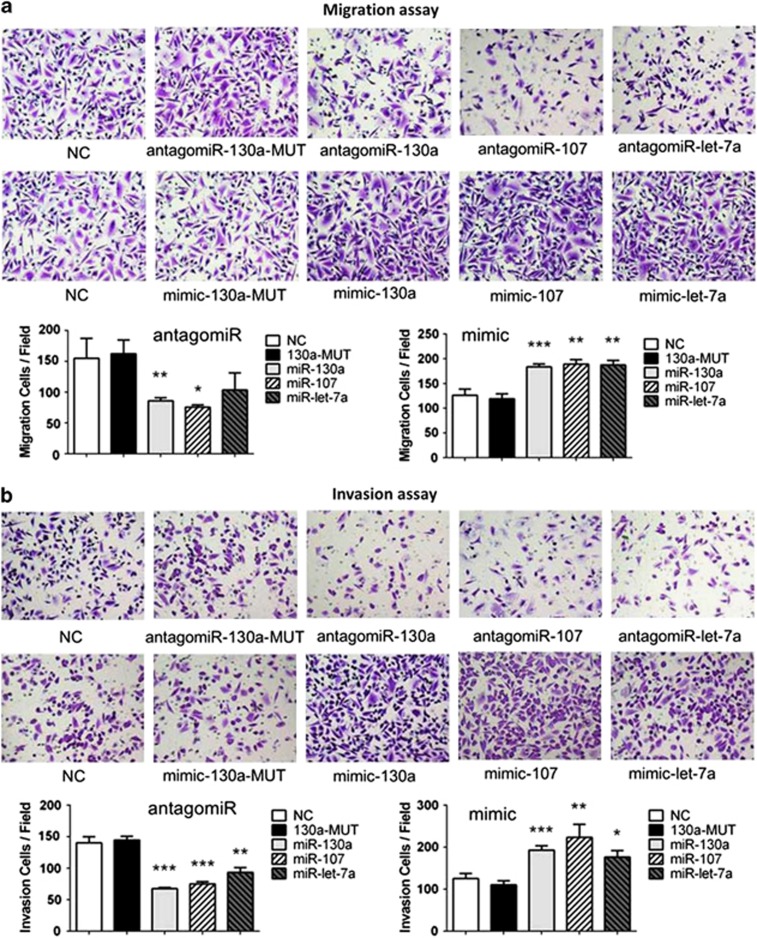
Transwell migration assays (**a**) and Matrigel invasion assays (**b**) of SiHa cells infected with antagomiRs, mimics and controls. Images shown at a magnification of × 200. The *P* values were calculated using Student's *t*-test. *P<0.05 *versus* control; **P<0.01, ***P<0.001

To test our hypothesis that Dicer-targeted miRNAs might indirectly promote migration and invasion of cervical cancer cells, we employed a transwell assay to evaluate the effects of miR-130a expression on cell migration and invasion. Other Dicer-targeted miRNAs, let-7a and miR-107, were also tested. As shown in Figure 5a, miR-130a, let-7a and miR-107 mimics noticeably increased SiHa cell migration, while antagomiRs against miR-130a, let-7a and miR-107 significantly decreased migration. Consistent results were observed in the cell invasion assay (Figure 5b). This *in vitro* evidence corroborated the observation that low Dicer expression correlated with metastasis of cervical cancer.

### MiR-130a expression is associated with survival of patients with cervical cancer

**Figure 6:**
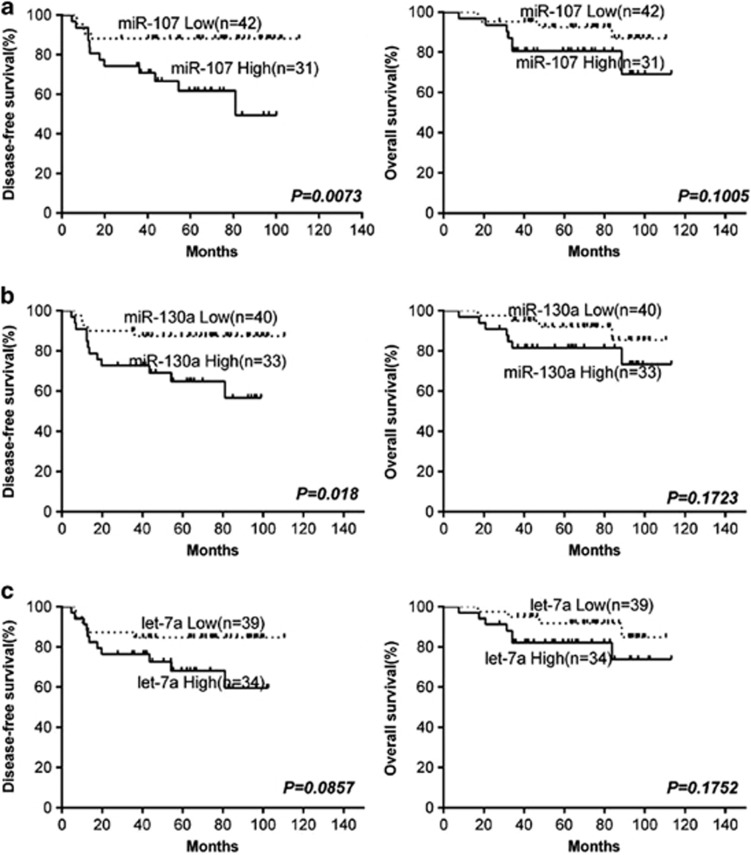
Kaplan-Meier graph representing the probability of disease-free survival and overall survival in cervical cancer patients (*n*=73) according to the miR-107 (**a**), miR-130a (**b**) and let-7a (**c**) relative expression. The log-rank test *P* value reflects the significance of the association between high miR-130a levels and metastasis

Based on the above findings, we hypothesized that Dicer-targeted miRNAs miR-130a, let-7a and miR-107 might also be associated with survival. We detected the expression levels of miR-130a, let-7a and miR-107 by qRT-PCR in 73 cases out of 102 patients whose RNA was available. We then performed survival analysis in comparison with the expression levels of these miRNAs. The DFS of patients with low miRNA-130a or miR-107 expression was higher than that of patients with high miRNA-130a or miR-107 expression (*P*=0.018, *P*=0.007, respectively; Figures 6a and b), and that of patients with low let-7a was marginally significantly higher than that of patients with high let-7a (*P*=0.086; Figure 6c). The OS of patients with low miRNA expression (miR-130a, let-7a and miR-107) was not significantly higher than that of patients with high miRNAs (*P*=0.172, *P*=0.175, *P*=0.101, respectively; Figures 6a–c). These clinical results were in agreement with biological functions of these miRNAs.

## Discussion

In this study, we found that low expression of Dicer mRNA and protein correlated with poor prognosis and relapse (including distance metastasis) of cervical cancer. Low Dicer expression was associated with patients with metastatic relapse. Low protein expression of Dicer was significantly associated with tumor stage. Patients with low Dicer mRNA and protein expression showed a shorter 5-year DFS and OS. Thus, low expression of Dicer seemed to be a significant prognostic factor for cervical cancer. These findings in cervical cancer are consistent with the results reported in the literature in other tumors.^5, 8^

Although Dicer mRNA and protein expression levels in cervical cancer were much lower than those in normal cervical tissue, the reduced Dicer mRNA in cervical cancer was not significant compared with normal cervical tissue. The lower expression level of the Dicer protein compared with that of Dicer mRNA in cervical cancer might be mediated by protein regulation; for example, reduced levels of the TRBP protein, an integral component of the DICER1-containing complex, resulted in a destabilization of the DICER1 protein.^22^

The literature suggests possible mechanisms underlying Dicer mRNA downregulation in cancer. The Dicer gene locus (on chromosome 14q) deletion might be one of the mechanisms in some cancers.^23, 24^ In addition, higher expression of miRNAs that target Dicer might be another critical mechanism, for example, miR103/107 were reported to target and downregulate Dicer expression and be associated with poor survival in breast cancer^15^ and gastric cancer.^25^ let-7 expression was inversely correlated with Dicer expression and constituted a negative feedback loop controlling Dicer expression in a panel of cancer cell lines.^16^ Interestingly, miR-107 can directly interact with let-7 and reduce its expression,^26^ suggesting a complex relationship between miR-107, let-7 and Dicer. More important, recent evidence shows that aberrant miRNA expression has important roles in cervical cancer.^27, 28^ These reports convinced us that miRNAs were involved in the low Dicer expression in cervical cancer. Therefore, we first identified 10 miRNAs that were predicted to target the 3′-UTR of Dicer transcript using several miRNA target prediction programs. qRT-PCR showed that low Dicer expression was correlated significantly and inversely with overexpression of the 10 miRNAs in cervical cancer. We then focused on miR-130a. Luciferase reporter assays with wild-type or mutated Dicer 3′-UTR, and cervical cancer cell gain- or loss-of-function assay with miR-130a mimics, mutants or antagomiRs indicated that miR-130a targeted Dicer directly.

In our study, miR-130a functions as an oncogene in cervical cancer, which is consistent with the data reported by Liu *et al.* in colon cancer^29^ and Wang *et al.* in NSCLC.^14^ However, some reports showed that miR-130a functioned as a tumor suppressor.^30, 31, 32^ The reasons for the apparently contradictory roles of miR-130a are not clear. One reason is that miR-130a may have different roles in different circumstances or tissues.

Recently, Su *et al.*^33^ found that tumor suppressor Tap63 could activate Dicer and miR-130b (one of the miR-130 family) at the promoter level to inhibit tumor metastasis. In contrast with the tumor suppressor role of miR-130b in Su's report, miR-130a was observed to have an oncogenic role in our study (i.e., it promotes cancer cell migration and invasion), suggesting that they might target different genes under different conditions. Surprisingly, however, we found that six miRNA-target prediction programs predicted Tap63 as a target of miR-130a and miR-130b, implying that miRNAs, Dicer and Tap63 might constitute a complex and accurate regulation circuit playing an important role in cancer development and progression.

In conclusion, we report, for the first time, that Dicer expression is an important prognostic factor in cervical cancer. Low Dicer expression is associated with decreased 5-year OS and DFS. Moreover, we present evidence that several miRNAs may affect Dicer expression. High expression of miRNA-130a attenuated Dicer expression. These results further increase our knowledge concerning the mechanisms of RNA interference. Finally, our findings may identify novel targets for the treatment of cervical cancer.

## Materials and Methods

### Cervical cancer samples and cell lines

This study was approved by the Review Board of our Cancer Center of Sun Yat-sen University. Formalin-fixed tissues were obtained from patients with invasive cervical cancer who underwent surgical resection at the Sun Yat-Sen University Cancer Center, Guangzhou, China, between 2002 and 2008. Ninety cancer tissues and 23 paired adjacent non-tumorous tissues were used for the qRT-PCR study. None of the patients had received chemotherapy or radiotherapy before surgery. One hundred and two paraffin-embedded tissues were used for immunohistochemistry, which included the above-mentioned 90 cancer tissues and another 12 patients who underwent cervical biopsy and accepted chemotherapy or radiotherapy. Thirty-four patients had paired adjacent non-tumorous tissues among the 102 paraffin-embedded tissues. Two pathologists diagnosed all the samples. The follow-up data in this study were available and complete. Histological types were assigned according to the WHO classification criteria. The 5-year DFS was 52.9%, with a median follow-up time of 5.27 years; the 5-year OS was 61.8%, with a median follow-up time of 5.70 years.

### Cell lines

The cervical cancer cell lines HeLa and SiHa were cultured in RPMI 1640 supplemented with 10% heat-inactivated fetal bovine serum (FBS) and 1% penicillin-streptomycin. All cells were incubated at 37 °C in a humidified chamber containing 5% CO_2_.

### RNA extraction and real-time PCR

Total RNA from cultured cells and fresh frozen tissues was extracted using Trizol reagent (Invitrogen, Carlsbad, CA, USA), according to the manufacturer's instructions. Reverse transcriptase reactions using MMLV reverse transcriptase reagents (Promega, Madison, WI, USA) were performed following the manufacturer's protocol. PCR conditions were 95 °C for 10 min; followed by 45 cycles of 95 °C for 30 s and 60 °C for 1 min. The primer pairs used for Dicer were 5′-ACACCTTTACCTGATGAACT-3′ and 5′-GTGTGGAATCTGAGGTATGG-3′, and for ACTB were 5′-ATGTGGCCGAGGACTTTGATT-3′ and 5′-AGTGGGGTGGCTTTTAGGATG-3′. For miRNA detection, reverse transcription followed by stem-loop qRT-PCR was performed according to the manufacturer's protocols, using the Bulge-LoopTM miRNA qRT-PCR Primer (RiboBio, Guangzhou, China). Real-time PCR was performed using Platinum SYBR Green qPCR SuperMix-UDG reagents (Invitrogen) in an Applied Biosystems PRISM 7900HT instrument. Expression variations were calculated using the 2^−ΔΔCt^ method. Total RNA input was normalized based on threshold cycle (Ct) values of common internal control for miRNA quantification assays, U6 snRNA, and all Ct values ≥36, which were considered as not expressed, were adjusted to 36.

### Immunohistochemistry

Immunohistochemistry was performed according to standard methods, as previously described.^34^ The anti-Dicer antibody used for the staining was a mouse monoclonal antibody (at a dilution of 1:400) (Abcam, Cambridge, UK). Dicer staining was observed in the cytoplasm. Control samples were stained in parallel, but were not incubated with either primary or secondary antibodies. The intensity of staining was graded on a scale from 0 to 3. Slides given a score of 0 represented no immunoreactivity, and a score of 3 represented strong immunostaining. Two pathologists confirmed the results in a double-blind analysis. Scores <2 were recorded as negative expression, while scores ≥2 were recorded as positive expression.

### MiRNA target prediction

*In silico* prediction of miRNAs that might target Dicer was performed using the algorithms PicTar (http://pictar.mdc-berlin.de/),^35^ TargetScan (http://www.targetscan.org/)^36^ and miRanda (http://www.microrna.org/microrna/);^37^ Venn diagram analysis was then performed to identify miRNAs that were conserved in three species.

### Transfection

MiRNA mimics were transfected into cells at a final concentration of 50 nmol/l using Lipofectamine RNAi MAX reagent (Invitrogen). Cells were incubated with miRNA mimics and appropriate scramble controls (all from GenePharma Company, Shanghai, China) for 4 h in Opti-MEM media before the addition of normal growth medium. The cells were then assayed for 48 h after transfection.

### Western blot analysis

Western blotting was performed according to standard methods (for details, see Supplementary Methods), using an anti-DICER antibody (1:400; Abcam, Cambridge, UK) as the primary antibody.

### Luciferase reporter assay

Briefly, 50 000 cells were seeded in one well of a six-well plate, in triplicate, and allowed to settle for 12 h. 293 T cells were transfected with 100 ng E-box reporter-luciferase plasmid or 100 ng control-luciferase plasmid plus 10 ng pRL-TK renilla plasmid using the Lipofectamine 2000 reagent (Invitrogen). Media were replaced at 6 h, and the luciferase and the renilla signals were measured 48 h after transfection using the Dual Luciferase Reporter Assay Kit (Promega Corporation, Beijing, China), according to the manufacturer's protocol.

### Cell migration assay and invasion assay

For the transwell migration assay, 2 × 10^5^ cells were placed in the top chamber of each insert (BD, Durham, NC, USA), without matrigel coating. For the invasion assay, 2 × 10^5^ cells were placed on the upper chamber of each insert, which was coated with 0.5 mg/ml Matrix gel Basement Membrane Matrix (BD) (for details, see Supplementary Methods).

### Statistical analysis

The best cutoff value for separating two groups in terms of gene expression levels (log2 of Dicer) was determined by Student's *t*-test. The association between various clinical characteristics and expression levels of Dicer was examined by the chi-square test or by Fisher's exact test. DFS and OS were estimated with the Kaplan–Meier method, and were compared by log-rank test using GraphPad Prism software (version5, GraphPad Software, La Jolla, CA, USA). Cox regression analysis was used to assess factors related to survival. All statistical analyses were performed with SPSS 16.0 software (SPSS Inc, Chicago, IL, USA). A *P* value<0.05 was considered significant.

## Figures and Tables

**Table 1:** Correlations between Dicer expression and clinicopathological features of patients with cervical carcinoma

**Characteristics**	**IHC data set (*****n*****=102)**			**qRT-PCR data set (*****n*****=90)**		
	**DICER**			**DICER**		
	**High**	**Low**	P*	**High**	**Low**	P*
*Age (years)*						
>35	36	45	0.088	43	26	0.8
⩽35	14	7		14	7	
						
*Tumor size*						
⩾4 cm	23	28	0.553	26	17	0.664
<4 cm	27	24		31	16	
						
*SCC*						
⩾1.5 ng/ml	10	18	0.125	14	9	0.806
<1.5 ng/ml	40	34		43	24	
						
*Histology*						
Adenocarcinoma	2	4	0.678	2	3	1
Squamous	48	48		54	31	
						
*Tumor grade*						
G1	17	17	1.000	20	11	1
G2/G3	33	35		37	22	
						
*FIGO stage*						
I–II	48	41	0.015	57	31	
III–IV	2	11		0	2	
						
*Lymph node metastasis*						
Yes	14	8	0.335	13	9	0.8
No	36	35		44	24	
Missing data	0	9				
						
*Distant metastasis and recurrence*						
Yes	7	19	0.012	7	14	0.002
No	43	33		50	19	

Abbreviations: FIGO, International Federation of Gynecology and Obstetrics; SCC, squamous cell carcinoma antigen

P*: *P*-values were calculated with a two-tailed Fisher's exact test

**Table 2:** Cox regression analysis of factors associated with disease-free survival and overall survival in cervical cancer patients

**Variables**	**Favorable/Unfavorable**	**IHC data sets**		**Real-time PCR data sets**	
		**HR (95% CI)**	***P***	**HR (95% CI)**	***P***
*Disease-free survival*					
Univariables					
Age	>35/⩽35	1.157 (0.464–2.885)	0.754	1.322 (0.512–3.413)	0.564
SCC	<1.5 ng/ml/⩾1.5 ng/ml	1.181 (0.513–2.718)	0.695	1.171 (0.454–3.02)	0.744
Tumor size	<4 cm/⩾4 cm	0.955 (0.443–2.061)	0.907	1.16 (0.492–2.732)	0.599
Histology	Squamous/adenocarcinoma	1.377 (0.324–5.84)	0.665	1.985 (0.458–8.607)	0.177
FIGO Stage	I, II/III ,IV	3.662 (1.582–8.475)	0.002	4.427 (1.277–15.342)	0.019
Tumor grade	G1/G2 or G3	1.296 (0.305–5.506)	0.755	0.998 (0.402–2.477)	0.36
Lymph node metastasis	No/yes	1.588 (0.64–3.939)	0.319	1.505 (0.607–3.735)	0.996
Dicer	High/low	2.859 (1.201–6.804)	0.018	4.105 (1.652–10.197)	0.002
Multivariate analysis					
FIGO Stage	I, II/III, IV	5.595 (1.302–14.036)	0.021	2.844 (0.8–10.106)	0.106
Dicer	High/low	2.969 (1.122–7.859)	0.072	3.715 (1.467–9.403)	0.006
*Overall survival*					
Univariables					
Age	>35/⩽35	1.046 (0.34–3.218)	0.937	1.461 (0.438–4.872)	0.538
SCC	<1.5 ng/ml/⩾1.5 ng/ml	2.126 (0.808–5.596)	0.127	1.641 (0.493–5.457)	0.419
Tumor size	<4 cm/⩾4 cm	1.474 (0.561–3.874)	0.432	2.286 (0.688–7.596)	0.893
Histology	Squamous/adenocarcinoma	2.472 (0.562–10.872)	0.231	4.736 (0.996–22.515)	0.177
FIGO Stage	I, II/III, IV	6.794 (2.548–18.117)	0	7.761 (1.626–37.046)	0.01
Tumor grade	G1/G2 or G3	2.004 (0.263–15.245)	0.37	1.486 (0.47–4.7)	0.051
Lymph node metastasis	No/yes	2.279 (0.721–7.208)	0.161	2.152 (0.68–6.814)	0.501
Dicer	High/low	3.418 (1.114–10.489)	0.032	5.794 (1.568–21.415)	0.008
					
Multivariate analysis					
FIGO Stage	I, II/III, IV	10.742 (1.638–23.726)	0.002	4.982 (1.019–24.371)	0.047
Dicer	High/low	2.346 (0.757–7.272)	0.152	5.13 (1.363–19.307)	0.016

Abbreviations: CI, confidence interval; HR, hazard ratio

## Abbreviations

siRNA: short interfering RNA
3′-UTR: 3′-untranslated region
NSCLC: non-small cell lung cancer
ROC curve: receiver operating characteristic curve
IHC: immunohistochemistry
qRT-PCR: quantitative real-time polymerase chain reaction
SCC: squamous cell carcinoma antigen
OS: overall survival
DFS: disease-free survival
HR: hazard ratio
NC: negative control
BC: blank control
TRBP protein: the TAR-binding protein
WHO classification criteria: World Health Organization classification criteria
RPMI 1640: Roswell Park Memorial Institute 1640
FBS: fetal bovine serum
Ct: threshold cycle
FIGO Stage: Federation of Gynecology and Obstetrics Stage
CI: Confidence Interval
