# Supplementary figures and images for: Prognostic significance of low DICER expression regulated by miR-130a in cervical cancer
Source: Cell Death Dis. 2014 May 1;5(5):e1205–. doi: 10.1038/cddis.2014.127 (PMC4047899; doi:10.1038/cddis.2014.127)

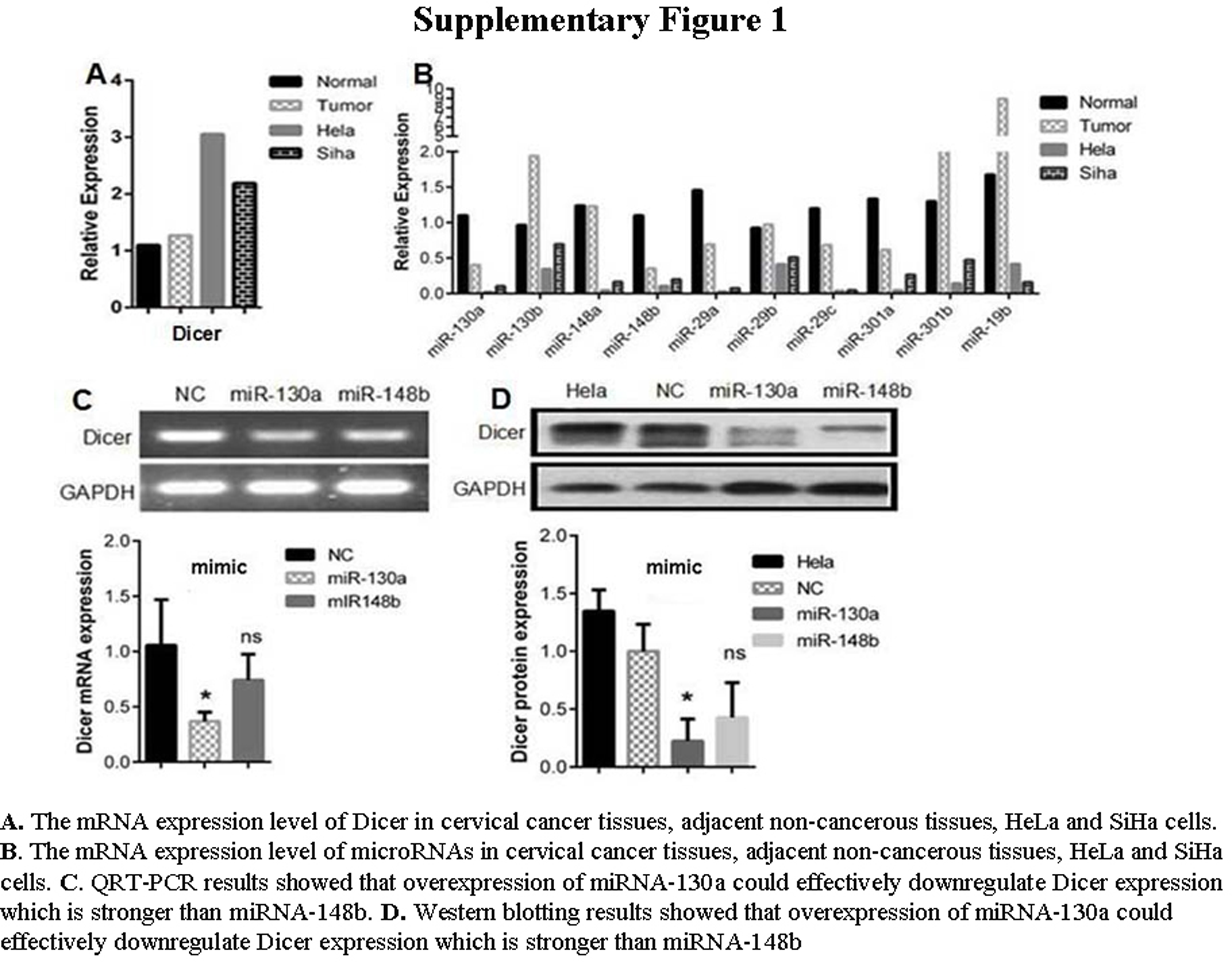

Supplement: Supplementary Figure 1 [file cddis2014127x1.tif]
