# Supplementary material for: Prognostic significance of low DICER expression regulated by miR-130a in cervical cancer
Source: Cell Death Dis. 2014 May 1;5(5):e1205–. doi: 10.1038/cddis.2014.127 (PMC4047899; doi:10.1038/cddis.2014.127)
Supplement: Supplementary Information [file cddis2014127x2.doc]

***Supplementary Methods***

***Western blot analysis***

Samples were frozen and lysed in buffer. The bicinchoninic acid (BCA) kit determined the protein concentration of each lysate according to the manufacture's protocol. Total protein (30 μg) was applied to each lane on 8% SDS-polyacrylamide gels. After electrophoresis and protein transfer, the polyvinylidene fluoride (PVDF) membranes were washed in Tris-buffered saline containing 0.05% Tween-20 and then incubated with anti-Dicer antibody (1:400) (Abcam, Cambridge, UK), as the primary antibody. The membrane was incubated with secondary antibody (1:2000) (Immunology Consultants Laboratory, Portland, OR, USA) and the ECL western blot analysis system was used to detect antibody binding, according to the manufacturer's protocol. ImageJ software (Image Processing and Analysis in Java) performed the quantification of the western blotting data.

***Cell migration assay and invasion assay***

For the transwell migration assay, 2×10^5^ cells were placed in the top chamber of each insert (BD Biosciences, Franklin Lakes, NJ, USA) without matrigel coating. For the invasion assay, 2×10^5^ cells were placed on the upper chamber of each insert, which was coated with 0.5mg/ml Matrixgel Basement Membrane Matrix (BD Biosciences). Both of the assays were conducted at 48h after the SiHa cells were transfected. Medium supplemented (700µL) with 10% fetal bovine serum was injected into the lower chambers. The cells were incubated at 37°C as follows: 8h for the migration assays; and 48h for the invasion assays. The insert membranes were fixed with 75% methanol for 20min. The cells on the upper surface were removed, and the migrated cells on the lower surface were stained with 0.5% crystal violet containing 20% methanol. The stained cells were counted under a microscope. Both experiments were repeated in triplicate, independently.
